# Concatenated CNN-Based Pneumonia Detection Using a Fuzzy-Enhanced Dataset

**DOI:** 10.3390/s24206750

**Published:** 2024-10-21

**Authors:** Abror Shavkatovich Buriboev, Dilnoz Muhamediyeva, Holida Primova, Djamshid Sultanov, Komil Tashev, Heung Seok Jeon

**Affiliations:** 1 School of Computing, Department of AI-Software, Gachon University, Seongnam-si 13306, Republic of Korea; abror1989@gachon.ac.kr; 2 Department of Infocommunication Engineering, Tashkent University of Information Technologies, Tashkent 100084, Uzbekistan; djamshidsultanov05@gmail.com (D.S.); k.tashev@tuit.uz (K.T.); 3 Tashkent Institute of Irrigation and Agricultural Mechanization Engineers, National Research University, Tashkent 100000, Uzbekistan; dilnoz134@rambler.ru; 4 Department of IT, Samarkand Branch of Tashkent University of Information Technologies, Samarkand 140100, Uzbekistan; primova@samtuit.uz; 5 Department of Computer Engineering, Konkuk University, Chungju 27478, Republic of Korea

**Keywords:** pneumonia detection, concatenated CNN, image enhancement, chest X-ray image

## Abstract

Pneumonia is a form of acute respiratory infection affecting the lungs. Symptoms of viral and bacterial pneumonia are similar. Rapid diagnosis of the disease is difficult, since polymerase chain reaction-based methods, which have the greatest reliability, provide results in a few hours, while ensuring high requirements for compliance with the analysis technology and professionalism of the personnel. This study proposed a Concatenated CNN model for pneumonia detection combined with a fuzzy logic-based image improvement method. The fuzzy logic-based image enhancement process is based on a new fuzzification refinement algorithm, with significantly improved image quality and feature extraction for the CCNN model. Four datasets, original and upgraded images utilizing fuzzy entropy, standard deviation, and histogram equalization, were utilized to train the algorithm. The CCNN’s performance was demonstrated to be significantly improved by the upgraded datasets, with the fuzzy entropy-added dataset producing the best results. The suggested CCNN attained remarkable classification metrics, including 98.9% accuracy, 99.3% precision, 99.8% F1-score, and 99.6% recall. Experimental comparisons showed that the fuzzy logic-based enhancement worked significantly better than traditional image enhancement methods, resulting in higher diagnostic precision. This study demonstrates how well deep learning models and sophisticated image enhancement techniques work together to analyze medical images.

## 1. Introduction

Pneumonia is a pathological process in the lungs, inflammation of the lung tissue. Many pathogens cause pneumonia: various viruses, bacteria, and fungi. As a result of the inflammatory process and the immune response, the normal functioning of the lungs is disrupted. Due to impaired gas exchange, the general condition of the body is suppressed, which can lead to death. Particularly dangerous is so-called atypical pneumonia, in which typical symptoms are expressed much less, and secondary symptoms prevail—sore throat, muscles, headache, and general weakness. It was atypical pneumonia, whose causative agent was the SARS-CoV-2 coronavirus, that was the main cause of death during the global pandemic that began in 2019. At the first stage of diagnosing respiratory diseases, the doctor must solve the problem of distinguishing between “normal” and “pneumonia”. In order to achieve this, the patient is put through radiation diagnostics, and radiography data are initially used to fix the issue.

Pneumonia has always been a hazardous disease, but after the emergence of the COVID-19 virus, the problem of its detection has moved to a new level. After all, if this disease is neglected and detected at a late stage, the consequences for human health or even life can be unpredictable. Tomography, X-ray, spirograph, bronchoscope, and other instruments are used to detect pneumonia, but X-ray is the most accessible of them; radiography is a quick way to detect pulmonary diseases in patients. The signs of pulmonary diseases are not always easy to detect and because of this, the analysis of only X-ray images can take a long time. Furthermore, because of COVID-19, the number of patients has grown, which has increased the strain on doctors. In order to promptly diagnose patients, they now need to review a vast amount of study data. This is time-consuming and can lead to errors, so to solve these problems, neural networks can be used to diagnose patients. Convolutional neural networks are the best tool for diagnosing diseases from medical images [1,2]. The main problems of using neural networks are increasing the accuracy and generalizing ability of networks, i.e., their ability to show high results not only on the data on which the network was trained but also on new data. The study aims to develop a CNN model for diagnosing pneumonia from X-ray images which has high accuracy and generalization ability.

In response to the limitations of traditional image enhancement techniques and CNN models, this study introduces a novel hybrid approach that combines fuzzy logic-based image enhancement with a multi-branch Concatenated CNN (CCNN) architecture. This combination significantly improves classification accuracy, precision, and robustness, particularly in medical imaging scenarios with variable image quality

The main contributions of our research are enumerated in the following list:

- Two neural network models are proposed for pneumonia detection: a traditional CNN and a novel Concatenated CNN (CCNN), trained on large-scale chest X-ray datasets comprising 5856 and 17,568 images, respectively. The CCNN leverages multiple image enhancement techniques for superior diagnostic performance;
- A novel fuzzy enhancement model is developed to significantly improve image quality during pre-processing. This method optimizes image contrast and highlights fine-grained details, making the diagnostic process more accurate and reliable;
- The quality of both the enhanced and original datasets is rigorously evaluated using the BRISQUE algorithm, providing a comprehensive comparison of image quality improvements;
- The performance of the proposed models is extensively compared against state-of-the-art architectures, including MobileNetV2, LSTM-CNN, AlexNet, ResNet-50, MobileNet, and VGG-19. This comparison is based on key metrics such as accuracy, precision, recall, F1-score, and AUC, demonstrating the superior effectiveness of our approach.

The following are the remaining sections of the paper: the relevant work is displayed in Section 2, the proposed image enhancement approach and CNN models are presented in Section 3, the experiments, results, and discussion are detailed in Section 4, and the conclusions are described in Section 5.

## 2. Related Works

Chest radiography has several benefits for detecting pneumonia, but one drawback is the shortage of qualified radiologists who can analyze the data, depict it for screening, and determine the illness’s severity. Thus, the creation and application of automated clinical decision support systems can greatly enhance the treatment of numerous patients by helping radiologists expedite the viewing and interpretation of data [3,4]. Artificial intelligence and deep learning are currently the most advanced methods for analyzing big data in almost all fields. Artificial intelligence-based computer systems have shown significant advances in the field of healthcare, and their use will significantly reduce the time to identify patients infected with the lung virus [5].

Currently, there is extensive research to identify an accurate and robust deep-learning model for pneumonia detection and classification. Researchers classify chest X-rays and CT scans of patients using various deep-learning models. The most commonly used method to solve this problem is the use of deep convolutional neural networks. Typically, these works solve the binary classification problem for the classes (normal and pneumonia) and obtain fairly high accuracy rates for pneumonia detection.

### 2.1. Artificial Intelligence and COVID-19 Using Chest CT Scan and Chest X-Ray Images

To strengthen the related work section with relevant references in the domain of artificial intelligence (AI) applied to medical imaging for COVID-19 diagnosis and treatment, recent research has explored various machine learning (ML) and deep learning (DL) techniques. Notable works include the use of chest CT scans and X-ray images for diagnosing COVID-19, the development of models to assist in vaccination strategies, and innovative pre-training methods guided by clinical priors. A comprehensive review of artificial intelligence (AI) applications for COVID-19 diagnosis using chest X-ray (CXR) and computed tomography (CT) images highlights significant advancements in AI-based methods for detecting the disease [6]. The study reviewed 22 papers published between January 2019 and June 2021, focusing on machine learning and deep learning approaches. The results showed that AI models achieved high performance, with an average accuracy of 93.7% for CXR and 89.1% for CT. Despite variability in results, especially for CXR, AI’s potential for disease monitoring, outbreak prediction, and management was evident. No significant accuracy difference was found between CXR and CT modalities. Cheng et al. [7] proposed a dynamic social network-based approach for optimizing COVID-19 vaccination strategies, especially in cases of limited vaccine supply. This method uses data assimilation techniques to update contact networks in real time, accounting for the variability in interactions and the impact of SARS-CoV-2 mutations. By identifying individuals with high centrality and degree in the network, the approach prioritizes them for vaccination. Tested on real-world and synthetic networks, it outperformed traditional methods in vaccination effectiveness, offering a more efficient strategy for targeting high-risk populations. Liu et al. [8] proposed advancements in medical vision–language pre-training (VLP); the IMITATE framework introduced a clinical prior guided approach for aligning hierarchical structures within medical reports and chest X-ray (CXR) images. Unlike previous methods that treat clinical reports as unified or fragmented entities, IMITATE leverages the inherent structure by aligning descriptive (“findings”) and conclusive (“impressions”) content separately with multi-level visual features. Additionally, a clinical-informed contrastive loss is proposed to enhance cross-modal learning. IMITATE outperforms traditional VLP methods across multiple datasets, demonstrating the effectiveness of integrating structured medical reports in vision-language alignment.

The authors of [9] proposed various convolutional neural network (CNN) models that were used as binary classifiers for lung X-rays and achieved 98% classification accuracy. The authors of [10] classified lung X-rays of healthy individuals and those with signs of COVID-19 infection using deep pre-trained CNN models ResNet50, ResNet18, ResNet101, VGG19, and VGG16. The ResNet50 model achieved an accuracy of 92.6%, while the deep learning CNN model developed in the paper achieved an accuracy of 91.6%. The authors of [11] proposed a deep learning DarkNet model for two classes, for which it obtained an accuracy of 98.08%, and for multiple classes of images, it achieved an accuracy of 87%. In [12], the authors proposed a convolutional neural network model to classify the Normal, Pneumonia, and COVID-19 classes with an accuracy of 92.4%. Similar studies also used various deep learning models pre-trained on large datasets, typically on the ImageNet dataset [13,14]. H. Nasiri and S. Hasani [13] used the DenseNet169 model to extract features from X-ray images and the XGBoost model to classify them. Thus, the authors obtained accuracy rates of 98.24% and 89.70% for two and three classes of X-ray images, respectively. In [14], the authors applied the Transfer Learning method to recognize pneumonia images and achieved a classification accuracy of 92.32%, precision is 95.69%, and recall is 95.62% for the pre-trained ResNet50 model. In [15], two-class classifiers (COVID-19, Normal) were implemented based on deep models using the 5-fold cross-validation method. The classification accuracy estimates obtained in the article allowed the authors to claim that the pre-trained ResNet152 model provided a classification accuracy of 96.1% among the other deep learning models considered in the article. H. Nasiri and S. Alavi [16] proposed a deep neural network using the ANOVA method for feature selection and subsequent binary classification, which achieved accuracy metrics of 92%. The authors of [17] proposed the CovXR model based on a deep convolutional neural network and obtained a classification accuracy of 95% for two classes of X-ray images. The authors of [18] developed several deep learning model architectures that were used to detect COVID-19, such as ResNet50, InceptionV3, VGG19, and others for classifying two classes of X-ray images. The best model with an accuracy of 98% in the article was recognized as the model based on the ResNet50 deep neural network.

The increase in COVID-19 CT scans and X-rays accessible since 2021 has caused research to move its attention to machine learning model comparison and development, as well as the creation of specific models for COVID-19 detection. Twenty CNN models, including EfficientNet-B5, DenseNet169, InceptionV3, ResNet-50, and VGG16, were evaluated using 4173 CT images in research by Garg et al. [19]. VGG-19 ranked lowest, with ResNet-50 and EfficientNet-B5 regularly outperforming the others in terms of sensitivity and accuracy. Chouat et al. [20] compared ResNet-50, InceptionV3, VGG-19, and Xcep-tion and obtained contrasting results. Xception scored best on X-ray pictures (98% accuracy), while VGG-19 excelled on CT scans (87% accuracy). Similarly, ResNet50 led in another CT imaging study [21] with a 96.97% accuracy rate, followed closely by Xception, InceptionV3, and VGG16. The performance of CNN models varies; hence, researchers have looked at hybrid or ensemble models that combine many networks. Combining Inception V3 and VGG16 allowed Srinivas et al. [22] to reach 98% accuracy in COVID-19 prediction, outperforming both models alone. Using a confidence fusion approach, Wang et al. [23] used characteristics from Xception, MobileNetV2, and NasNetMobile to achieve classification.

Research findings indicated a range of classification accuracy, from 78% to 100%, which might be explained by the quantity or caliber of the dataset. Karar et al. [24] used a small dataset of 263 original X-ray pictures; however, they claimed 99.9% accuracy for VGG-19 and ResNet-50. In contrast, using a larger dataset of 4326 chest X-ray pictures, Kumar et al. [25] obtained 100% accuracy for binary classification (normal vs. COVID-19) and 98.82% accuracy for multi-class classification (normal, COVID-19, pneumonia).

### 2.2. Fuzzy Pre-Processing Models in Deep Learning for Pneumonia Detection

The performance of deep learning (DL) models in medical image classification, particularly in diagnosing diseases like pneumonia, is heavily influenced by the quality of image pre-processing methods. Researchers have demonstrated that sophisticated pre-processing techniques can significantly enhance model performance by improving image clarity and reducing noise [26,27,28]. One such approach is the use of fuzzy logic, which has been increasingly integrated into deep learning models to improve the accuracy of medical diagnoses.

Cosimo et al. [29] introduced a fuzzy logic-based deep learning model specifically for pneumonia detection. In their model, a fuzzy edge detection algorithm is applied during the pre-processing phase. This technique aims to sharpen the edges of anatomical structures within chest X-rays, making it easier for convolutional neural networks (CNNs) to detect anomalies such as pneumonia. Traditional edge detection methods can often struggle with medical images due to the presence of noise or varying tissue densities, but fuzzy logic allows for a more nuanced interpretation of image boundaries, enhancing the overall quality of the input data. Another innovative approach was proposed by Shin et al. [30], who developed a fuzzy logic histogram equalization algorithm to enhance chest X-ray images. This method adjusts the contrast of medical images using fuzzy membership functions, making important features such as lung opacities more distinguishable. Histogram equalization typically results in better global contrast but can sometimes cause a loss of detail in subtle areas. Fuzzy logic, however, enables more adaptive contrast adjustments, preserving finer details while enhancing the overall visibility of critical structures. In [31], the authors further developed this concept by employing fuzzy logic to improve X-ray images in a different way. They designed an algorithm that reduces background noise and optimizes pixel intensity variation based on a fuzzy membership function. This technique allows for more precise image enhancements than traditional pre-processing approaches, which often treat all pixels uniformly. By assigning fuzzy membership values based on intensity and noise thresholds, this method enhances the quality of the input data, leading to better performance in subsequent deep learning model classification. Additionally, Sousa et al. [32] contributed to the field by using fuzzy divergence to select the optimal gray tone for enhancing medical images. This method calculates the fuzzy divergence of various tones and applies enhancement through fuzzy membership values. This process leads to a clearer distinction between normal and pathological regions in chest X-rays, which is crucial for models that rely on pixel-level distinctions to make accurate predictions.

Despite the clear benefits of using fuzzy logic in pre-processing, CNN-based transfer learning models sometimes struggle with overfitting, particularly in the testing phase, even when they show high accuracy during training and validation. This is due to the inherent limitations of CNN models, which are often pre-trained on large datasets such as ImageNet. These datasets may not provide enough discriminating features that are specific to medical images, which tend to have different characteristics than the everyday images used in general-purpose datasets. Consequently, when these models are fine-tuned for medical image classification, they may retain features that are not well-suited for distinguishing between medical conditions like pneumonia and normal lung tissue [33,34,35,36,37,38,39,40,41].

Fuzzy logic-based pre-processing methods, however, can help mitigate some of these challenges by providing clearer, more refined input data. The better the quality of the input, the less likely the model is to overfit to irrelevant features. By reducing noise, optimizing pixel intensity, and enhancing the clarity of important structures, fuzzy logic techniques contribute to the robustness and accuracy of deep learning models, making them more reliable for real-world medical applications.

## 3. Proposed Method

**Figure 1:**
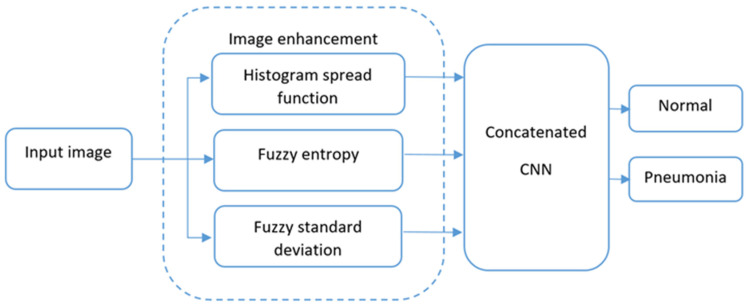
The block-scheme of the proposed model.

To accurately detect pneumonia, the study combined the advantages of fuzzy techniques and deep learning into one model, i.e., fuzzy logic with local contrast analysis ability and feature extraction, and the learning ability of deep learning models. The integrated method helped to improve the accuracy of pneumonia detection in contrast with previous deep learning models. Figure 1 illustrates the overall flowchart of the proposed model.

The proposed framework of the model has the following modules:

Input image: this block inputs X-ray images.

Image enhancement: this block uses a novel algorithm of fuzzification procedure to increase the quality of the images. This technique optimizes the fuzzification process by using a mathematical method to find the membership function. Furthermore, the block outputs three transformed images using the histogram spread, fuzzy entropy, and fuzzy standard deviation functions. The image enhancement algorithm consist of 12 steps.

Concatenated CNN model: The proposed Concatenated CNN model is trained on the fuzzy-enhanced dataset. The detailed information of the dataset development process is described in the next section. The CNN model gains the ability to distinguish between regions that are pneumonia and those that are not by using features extracted from the improved images.

Output: The output of the model is the detection of pneumonia or normal cases.

The proposed pneumonia detection model integrates the fuzzy data processing method for improvement of images with the benefits of the CNN models such as feature extraction, high accuracy, and learning capabilities.

The construction and training process of CNN model includes the following steps:

- Model Construction: Activation functions, dropout for regularization, and convolutional, pooling, and dense layers comprise the CNN architecture, which is defined by using Keras 2.13.1;
- Model Training: TensorFlow 2.15 and Keras are used to build the model, define the optimizer (Adam), and input metrics (accuracy) and loss function (binary cross-entropy). A GPU was used to speed up processing during the model’s training process.

The Anaconda 2020 Python distribution is used to build and test the recommended configuration on a computer with two Nvidia GeForce 1080Ti GPUs (Nvidia, Santa Clara, CA, USA), 32 GB of RAM, and a 3.20 GHz CPU.

### 3.1. Input Dataset and Image Enhancement Using Fuzzy Technique

Medical image datasets serve as the cornerstone of our research, facilitating the training and evaluation of neural networks for lung disease detection. A comprehensive dataset preprocessing includes several key steps, ensuring the diversity and representativeness required for robust model performance. In the context of medical imaging for pneumonia detection, intelligent sensors play a crucial role in acquiring high-quality data that form the foundation of deep learning models. The primary imaging modalities used in this study, such as chest X-rays and CT scans, rely on advanced sensor technology to capture high-resolution images. The quality and precision of these sensors directly impact the performance of computer-aided diagnostic systems, including the proposed Concatenated CNN model.

**Figure 2:**
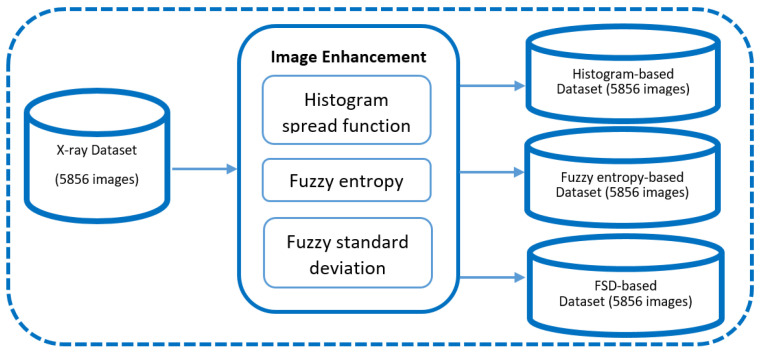
Dataset development process.

1. X-ray dataset: In the initial stage, a chest X-ray pneumonia detection dataset is used, which is publicly available on Kaggle [42]. The dataset has 5856 X-ray images, of which 1583 images were classified as “Normal” and 4273 images were labeled “Pneumonia”;

2. Image enhancement: The second critical step involves enhancing all 5856 images using a Fuzzy Inference System (FIS). This innovative approach introduces an additional layer of complexity by including different degrees of membership for each pixel in the image. The new algorithm is implemented in the fuzzy logic process to improve the image quality. In addition, a mathematical algorithm for refining the membership function was developed, facilitating the refinement of the fuzzy logic;

3. Generating transformed datasets: Using three types of local contrast features, three new datasets are obtained. Each dataset has 5856 FIS-enhanced images. After the fuzzy enhancement process, the number of images increased three times and the dataset contained 4749 normal images and 12,819 pneumonia images. A training set, testing set, and validation set were each allocated 75%, 15%, and 10% of the complete dataset, which comprised 17,568 images. This dataset encapsulates fine-grained representations of lung conditions after fuzzy logic processing, introducing elements of uncertainty and imprecision that are vital for a comprehensive assessment. See Figure 2.

#### Fuzzy Image Enhancement Technique

The image enhancement techniques employed in this study, such as fuzzy entropy and standard deviation-based enhancement, benefit directly from the high-fidelity data produced by modern sensors. These sensor-driven images allow for the extraction of fine-grained features, which are essential for improving the accuracy, precision, and robustness of the CCNN model in pneumonia detection.

The developed datasets serve as a training and testing ground for our developed convolutional neural networks, aimed at studying the impact of FIS-transformed images on model performance. As such, the data augmentation process is carefully designed to cover the spectrum of lung diseases, providing a robust foundation for training the neural networks. The integration of FIS enhancement introduces a unique dimension that is consistent with our overarching goal of improving neural network detection capabilities in the field of pneumonia detection.

**Figure 3:**
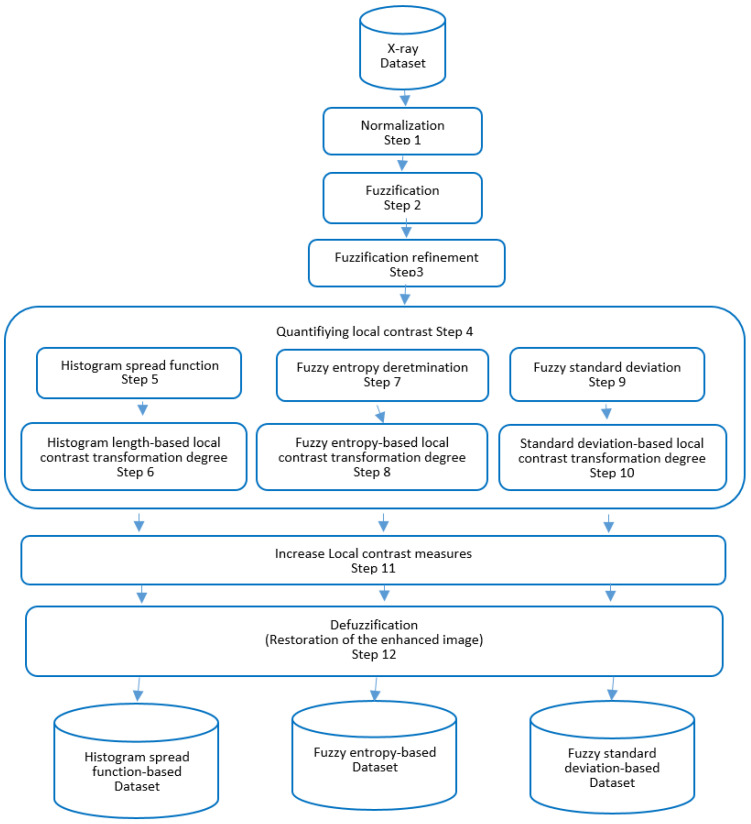
Image enhancement process using fuzzy techniques.

A fuzzy data augmentation algorithm is proposed to improve the image quality in the fuzzy logic process. This algorithm includes an advanced mathematical approach to determine the membership function, optimizing the fuzzy logic process. The resulting fuzzy-transformed images are a specialized dataset designed for training CNN. The image enhancement algorithm consists of 12 steps and is illustrated in Figure 3.

By giving each pixel in an image a varying degree of membership, the idea of fuzziness in image processing adds another level of complexity. This makes it possible to account for uncertainty in the description of things shown in a picture. An image “F” is represented in a fuzzy framework in which each pixel is linked to degrees of membership, which indicate how much the pixel belongs to certain groups or categories.

This method offers a more flexible and nuanced representation for studying and understanding images by acknowledging and accounting for the inherent ambiguity and imprecision frequently present in real-world images.

(1) $$F=\left\{\langle f(x,y),\;\;\mu_{F}(f(x,y)))\rangle \left|f(x,y)\in \{0,\ldots ,L-1\}\right.\right\},$$

where x∈{1,…,M}, y∈{1,…,N}, $\mu_{F}(f(x,y))$ denote, respectively, the degree of membership of the (x, y)-th pixel to the set in accordance with the properties of the image.

When handling image processing jobs that include ambiguity, fuzzy images tend to be quite useful. Fuzziness is sometimes useful for segmenting items in image, particularly when the exact borders of the objects are difficult to see. Because it finds it difficult to handle the underlying ambiguity of an image, standard binary image processing may be useless in some situations.

Step 1. Normalization:

(2) $$u(x,y)=l\frac{f(x,y)-f_{\min}}{f_{\max}-f_{\min}}.$$

Step 2. Fuzzification:

(3) $$\mu_{F}^{i}(x,y)=\frac{1}{1+\frac{u(x,y)\;-\;c_{i}}{\sigma_{f}}},\;\;\;i=\bar{1,k}.$$

Step 3. Fuzzification refinement:

(4) $$\mu_{F}^{i}(x,y)=\left\{\begin{array}{l} 2{\left(\mu_{F}^{i}(x,y)\right)}^{2},\;\;\;\;\;\;\;\;\;\;\;\;\;\;\;\;\;\;0\leq \mu_{F}^{i}(x,y)\leq \frac{1}{2},\\ 1-2{\left(1-\mu_{F}^{i}(x,y)\right)}^{2},\;\;\;\;\frac{1}{2}<\mu_{F}^{i}(x,y)\leq 1. \end{array}\right.$$

Step 4. In image processing, quantifying local contrast is essential because it makes evaluating contrast variations between various picture areas easier. Two distinct formulaic methods have been put forth to compute local contrast in the context of 8-bit grayscale digital pictures in order to aid in the precise assessment of contrast levels in a geographical region of an image. With the use of these approaches, contrast fluctuations in an image may be quantified, enabling more deliberate and focused image processing choices. The two formulae are shown as follows:

Calculations of local contrast:

(5) $$C(x,y)=(C_{\max}-C_{\min})/255$$

Calculations of global contrast:

(6) $$C\left(x,y\right)=\frac{{\left\{2\frac{\sum_{j=1}^{n}{\left[f_{j}\;-\;M\left[f_{j}\right]\right]}^{2}\mu_{j}}{\sum_{j=1}^{n}\mu_{j}}\right\}}^{0.5}}{255},$$

where $C_{max}$ and $C_{min}$ are the maximum and minimum brightness values in the vicinity of pixels.

The many local neighborhood categories, which are identified by varying degrees of pixel luminance smoothness, must be examined in order to fully understand local contrast and its applications in image processing:

- A homogeneous neighborhood is defined as a local neighborhood where the brightness values of the pixels are comparable or the same. This indicates a high level of homogeneity in the local neighborhood. In an image, the sky region or other regions where the pixel brightness is nearly constant would be examples of such a neighborhood. That is why there will not be any local contrast in this kind of area;
- A binary neighborhood is a local neighborhood characterized by elements displaying the extremes of the luminance spectrum; in this example, the local neighborhood consists of pixels, like black and white pixels, whose luminance values obviously occupy opposite ends of the range. These zones stand out for their extreme contrast, despite the possibility of abrupt shifts in brightness levels and non-uniformity;
- A local neighborhood including components of different brightness values: in this case, the local neighborhood covers pixels with different luminance values, although the transitions between them are not sharp or noticeable. These communities frequently include intricate features, varied textures, or a range of items with varying degrees of brightness, all of which lack distinct borders.

Comprehending the characteristics and makeup of nearby communities is crucial for calculating local contrast. This is because, in order to obtain the intended outcomes, various neighborhood types may need alternative techniques for contrast estimation or divergent processing settings to be used. Let us examine how various values of local features, such as entropy, the histogram distribution function, and standard deviation, may be used to discriminate between distinct neighborhood groupings. These characteristics are useful measurements for determining the position and contrast of particular areas within an image.

Step 5. This involves utilizing the Cumulative Distribution Function (CDF), which measures the percentage of pixels within an image that have brightness values that are either below or equal to a predetermined threshold.

(7) $$h_{F}(x,y)=\frac{f_{\max}-f_{\min}}{h_{\max}},$$

where $f_{max}$ and $f_{min}$ are the minimum and maximum brightness values in a sliding neighbourhood *W* centred on an element with coordinates (*x*, *y*); $h_{max}$ is the maximum histogram value of a sliding local neighbourhood *W* centred at an element with coordinates (*x*, *y*).

In homogeneous regions, this local neighborhood feature takes minimal values, whereas in binary areas, it reaches maximum values. Since most pixels in a homogenous region have identical brightness values, the CDF will show a pattern that is almost linear. Given the existence of two dominating brightness levels, the CDF in a neighborhood that is conditionally binary will exhibit noticeable steps. When there are neighborhoods with fluctuating brightness values and no abrupt changes in brightness, the CDF will show more gradual progression.

**Figure 4:**
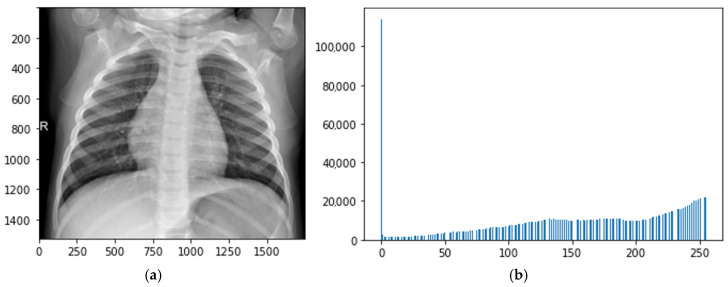

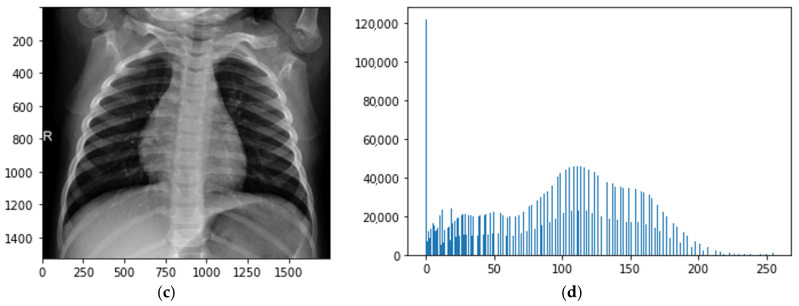
Transformations of local contrasts based on histogram length functions: (**a**) original image; (**b**) histogram of original image; (**c**) defuzzified image; (**d**) histogram of defuzzified image.

Step 6: This uses histogram length functions to gauge how much local contrasts have been transformed:

(8) $$\alpha =(\alpha_{\min}-\alpha_{\max}){\left(1-\exp(-\frac{{\left(h_{F}-a\right)}^{2}}{2\pi^{2}})\right)}^{s},$$

where *s* > 0. See Figure 4.

Step 7: Entropy: an indicator of the degree of variation or uncertainty in pixel values within a neighborhood is entropy. An increased entropy score denotes a wider range of pixel intensities within the defined area. Because there is little variation in the pixel values inside a homogeneous neighborhood, which is made up of pixels with almost identical intensities, the entropy will be reported as low. The substantial variation of values in a basically binary neighborhood characterized by pixels with intensities at the extreme ends of the spectrum may result in a high entropy. The entropy may be in the moderate range in neighborhoods with varying intensities and smooth transitions between pixel values, indicating a moderate level of variability. The following expression defines fuzzy entropy in a sliding local region of size *n* × *m*:

(9) $$\varepsilon (\mu_{F})=-a\sum_{i=1}^{n}\{\mu_{F}(f_{i})\ln\mu_{F}(f_{i})+[1-\mu_{F}(f_{i})]\ln[1-\mu_{F}(f_{i})]\}/\log(nm),$$

where ${µ}_{F}(f_{i})$ is calculated as follows:

(10) $$\mu_{F}(f_{i})=h_{F}\left(f_{i}(x,y)\right)/\left(n\times m\right).$$

Here, the value of the histogram inside the local neighborhood represented by *W* is shown by *h_F_*($f_{i}$(*x*, *y*)). Specifically, it shows how many elements in this neighborhood *W* have brightness values f_i_ (x, y) that match the element at coordinates (*x*, *y*). Expression (9) states that regions with homogeneity have the highest fuzzy entropy values, whereas regions with components that display brightness values at opposing extremes of the spectrum have the lowest fuzzy entropy values.

**Figure 5:**
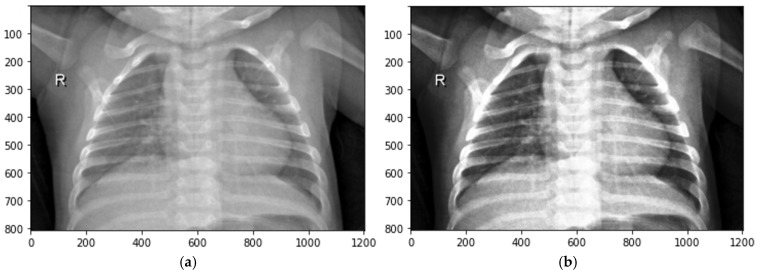
Transformation of local contrasts by fuzzy entropy: (**a**) original image; (**b**) defuzzified image.

Step 8: Use fuzzy entropy to determine how much local contrasts α have been transformed:

(11) $$\alpha =\alpha_{\min}+(\alpha_{\max}-\alpha_{\min}){\left(\frac{\varepsilon (\mu_{F})-\varepsilon_{\min}}{\varepsilon_{\max}-\varepsilon_{\min}}\right)}^{s},$$

where *s* > 0. See Figure 5.

Step 9: The standard deviation, typically denoted by the symbol σ (sigma), measures how brightness values are distributed or dispersed in the region of their mean. The standard deviation is often low in a homogenous area, where data are closely concentrated around the mean. On the other hand, if there is a large disparity between the minimum and maximum brightness values in a conditionally binary neighborhood, the standard deviation could increase. Neighborhoods with a range of brightness values and no noticeable transitions may have a moderate standard deviation. Analyzing these features aids in comprehending the contrast and structural properties of distinct picture areas. This knowledge is useful for selecting the best processing techniques and modifying processing plans in light of the distinctive features of nearby communities. Formula (12) is used to compute the standard deviation of brightness values of the components in a moving neighborhood W.

(12) $$\sigma {\left(x,y\right)}_{F}=\sqrt{\frac{1}{nm}\left(\frac{\sum_{j=1}^{n}{\left[f_{j}-M[f_{j}]\right]}^{2}\mu_{j}}{\sum_{j=1}^{n}\mu_{j}}\right)},$$

where $M[f_{i}]$ is fuzzy arithmetic mean value of the brightness of elements of a local neighbourhood W centred at element $M\left[f_{i}\left(x,y\right)\right]\;$with coordinates (*x*, *y*):

(13) $$M[f_{j}]=\frac{1}{NM}\sum_{x=1}^{N}\sum_{y=1}^{M}f_{j}(x,y),$$

where N, M are the dimensions of the $x=\bar{1,N},\;y=\bar{1,M}$ image.

In homogeneous neighborhoods, expression (12) equals zero and grows as heterogeneity increases.

**Figure 6:**
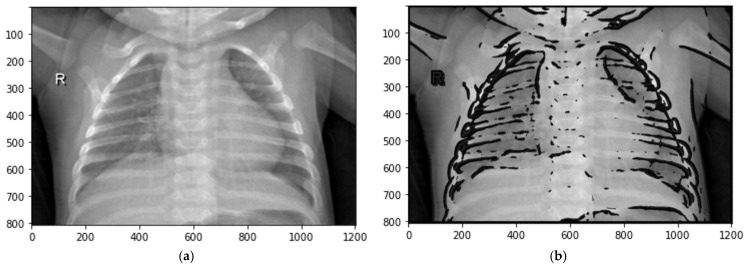
Transformations of local contrasts based on the fuzzy standard deviation of brightness values: (**a**) original image; (**b**) defuzzified image.

The fuzzy standard deviation of brightness data is used in Step 10 to ascertain the extent of local contrast change.

$$\alpha =\alpha_{\min}\sigma (x,y)+\alpha_{\max}(1-\sigma {\left(x,y\right)}_{F}^{s})$$

where *s* > 0. See Figure 6.

Step 11: Increase some quantitative measure of local contrast in accordance with a certain legislation. This phrase is utilized in relation to a nonlinear transformation applied to local contrast:

(14) $$C^{*}(x,y)=\left\{\begin{array}{l} B_{0}+\left(\frac{R}{2}-A_{0}\right){\left(\frac{C(x,y)-C_{\min}}{\overset{\wedge }{C}-C_{\min}}\right)}^{\alpha }\;\;\;\;\;\;\;\;\;\;\;\;\;\;\;\;\;\;\;\;\;\;\;\;\;\;\;\;\;\;C(x,y)\leq \overset{\wedge }{C,}\\ R-A_{0}-\left(\frac{R}{2}-A_{0}\right){\left(\frac{C_{\max}-C(x,y)}{C_{\max}-\overset{\wedge }{C}}\right)}^{\alpha }\;\;\;\;\;\;\;\;\;\;\;\;\;\;\;\;\;\;\;\;\;C(x,y)>\overset{\wedge }{C}, \end{array}\right.$$

where R is the highest feasible local contrast value R = 1, C(x, y) is the local contrast value of the original image element with coordinates (x, y), and C* (x, y) is the enhanced local contrast value of the image element with coordinates (x, y). The initials C_min_ and C_max_ stand for the highest and lowest local contrast levels in the original image, respectively. As an example, the arithmetic mean of the image components’ local contrasts is C*, which is an approximation of the mathematical expectation of the local contrast values; constant bias coefficients A_0_ and B_0_ are used. α is the exponent with α < 1.

Step 12: Using improved local contrast, reconstruct the changed image parts.

An essential first step in image processing is the design of a local contrast transform function. Its formulation is contingent upon some factors that the researchers have established, such the limitations that establish the variation in contrast enhancement degree. These boundaries are crucial in deciding how much local contrast will be improved in various areas of the image. The researcher’s expertise and knowledge of local statistical features are essential for choosing the contrast transform function’s parameters.

The selection of parameters is contingent upon the particular objectives and intended outcomes of image processing, as there is no generally applicable theoretical approach to attain optimal contrast transform. Finding a compromise between contrast improvement and artifact reduction is the primary objective when developing a transform function.

### 3.2. Architecture of Proposed CNN Models for Pneumonia Detection

CNN layers are the most crucial part of our suggested model. The goal of utilizing this component is to incorporate the benefits of CNN models, which are quick, effective, and enable accurate diagnosis of both normal and pneumonia cases. In this work, two types of deep learning models are developed, i.e., CNN for a dataset with 5856 enhanced images, and Concatenated CNN. The CCNN is trained on a dataset with overall 17,568 images. The CCNN model is used because after preprocessing, the fuzzy enhancement model outputs three images. Our pneumonia detection system’s CCNN and CNN models are described in the sections below.

#### 3.2.1. The Concatenated CNN Model

The CCNN model introduced in this work utilizes three distinct input branches for different image enhancement techniques—fuzzy entropy, standard deviation, and histogram equalization. This unique multi-branch design enables the model to leverage complementary features from each technique, resulting in significantly improved performance metrics across various datasets.

**Figure 7:**
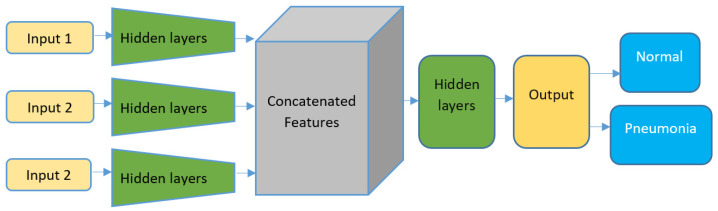
The architecture of the proposed CCNN model.

The design for a Concatenated CNN model with three input layers for pneumonia detection using 512 × 512 X-ray images is illustrated in Figure 7.

Input 1: First improved image, preprocessed using the histogram spread function.
Input 2: Second variant of improved image, preprocessed through the fuzzy entropy function.
Input 3: Third enhanced image, preprocessed using the fuzzy standard deviation function.

The detail information about architecture CCNN model is presented in Table 1.

#### 3.2.2. The CNN Model

**Figure 8:**
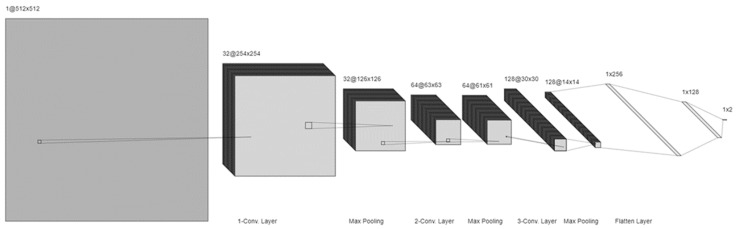
The architecture of the CNN model.

The CNN also utilized as feature extractor model as presented in Figure 8. Each of its several linked layers carries out a distinct task in the processing and analysis of visual data. Through a progressive process of extracting abstract elements from input images, the architecture enables the model to learn and detect patterns.

This section offers a thorough explanation of the training settings and model design.

Layer of Input: This layer accepts the enhanced images that were processed using the fuzzy enhancement model. The standard size of these input photos is 512 by 512 pixels, which was chosen to balance processing performance with preserving sufficient information for accurate classification.

Layers of Convolution: The activation function named ReLU (Rectified Linear Unit) follows 32 filters with a 3 × 3 kernel size in the first convolutional layer. This layer’s main goal is to extract basic components, such as edges and rudimentary textures, from the incoming pictures. The resulting feature map has dimensions of 254 × 254 × 32.

The ReLU activation function is used after 64 filters, each with a 3 × 3 kernel, in the second convolutional layer. This layer can capture more detailed information by building upon the findings of the preceding layer. The feature map’s size is reduced to 63 × 63 × 64 due to max pooling.

After 128 filters with a 3 × 3 kernel size, the third convolutional layer applies the ReLU activation function. By detecting high-level features such as specific patterns linked to pneumonia, this layer abstracts the visual data even further. The final size of the feature map is 30 × 30 × 128 after pooling.

Pooling Layers: Each convolutional layer is followed by a max pooling layer with a pool size of 2 × 2 and a stride of 2. These layers perform a downsampling of the feature maps, preserving the most significant features while reducing the computational load and spatial dimensions of the feature maps. This step is crucial for preventing overfitting and improving the model’s ability to generalize.

Fully Connected Layers: The first fully connected layer, also known as the initial completely networked layer, is made up of 256 neurons that ReLU has engaged. By transforming the 3D feature maps into a 1D feature vector, it enables the model to carry out complex feature interactions. This layer is necessary to merge the retrieved information into a more abstract representation.

The second fully connected layer, which serves to enhance feature extraction and prepare the data for the final classification layer, is made up of 128 neurons that ReLU has engaged.

Using a SoftMax activation function, the output layer assigns a probability score for binary categorization (normal or pneumonia). This layer generates a vector of two probabilities representing the likelihood of each class. Since the SoftMax function ensures that the probability sums up to 1, the model’s predictions make sense.

Binary Cross-Entropy is an appropriate loss function for binary classification applications.

The suggested CNN model’s training parameters are:

Optimizer: The Adam optimizer, renowned for its versatility and effectiveness in deep learning applications, has an initial learning rate of 0.001.

Batch Size: 32, which strikes a balance between convergence speed and processing burden.

Epochs: 50 epochs provide the model enough time to learn without overfitting.

Early Stopping: This feature was added to track the validation loss and stops training if the validation loss does not improve after 10 epochs of patience.

## 4. Experiments and Discussions

The performance of the model was experimented with different combinations of experiments. Comparing the concatenated convolutional neural network (CCNN) trained on the fuzzy-transformed dataset to its counterparts trained on the original image dataset, the testing findings showed notable improvements in pneumonia diagnosis. Furthermore, the CNN model is trained using a dataset that is improved using the traditional CLAHE method. An extensive evaluation using many quantitative metrics, such as accuracy, precision, AUC, F1-score, and recall, shows how effective the proposed method is.

The experiments examine the impact of different image enhancement methods on the performance of five convolutional neural network models designed for classification tasks. Four distinct kinds of datasets were used to train the CNN models: original images, and images enhanced by fuzzy standard deviation, fuzzy entropy, and histogram equalization. A concatenated model that included characteristics from all the upgraded datasets was also created. The goal is to investigate how various image enhancing techniques increase the resilience and accuracy of CNN classification.

The performance comparison of five measures (Accuracy, Precision, Recall, AUC, and F1-Score) for different pneumonia detection models is shown in Table 2. Accuracy of 87.1% was attained by the CNN trained on the original, unaltered pictures, with precision, recall, and F1-score in the mid-80% range. This model performed quite well; however, it had trouble extracting pertinent information for precise categorization. It appears from the relatively low AUC of 84.7% that the model struggled to discriminate between classes, which might mean that significant picture attributes were not adequately captured in their raw form.

**Figure 9:**
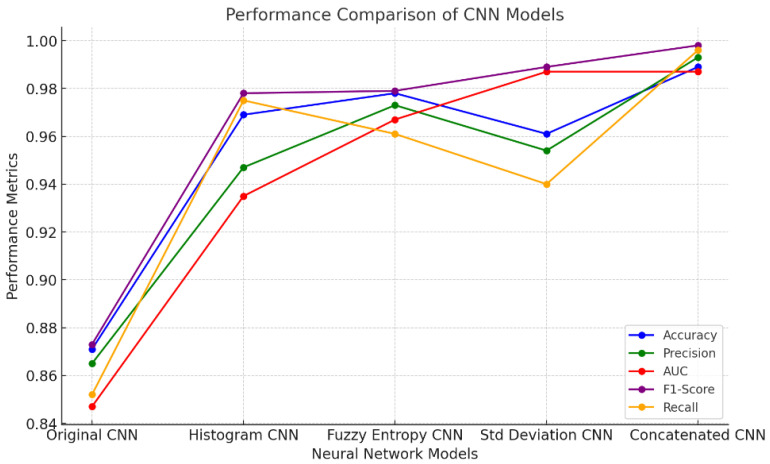
Comparison of performance of CNN models.

Three image improvement approaches (fuzzy entropy, standard deviation-based enhancement, and histogram equalization) were used to improve feature extraction. Figure 9 shows much more understandable information. With accuracy reaching 96.9%, the CNN trained on the histogram-equalized images performed noticeably better. By distributing pixel intensities more equally and enhancing contrast, this enhancement technique helped the model better capture global information. When compared to the initial image-based CNN, the precision (94.7%), recall (97.5%), and F1-score (97.8%) all showed significant improvements, and the AUC climbed to 93.5%, indicating greater class separation. With a 97.8% accuracy rate, the CNN that was trained on fuzzy entropy-enhanced images performed even better. Fuzzy entropy is a very useful technique for collecting fine-grained features since it highlights minute differences and complexity in the picture data. AUC was 96.7%, accuracy was 97.3%, recall was 96.1%, and the model had a strong F1-score of 97.9%. These findings suggest that the fuzzy entropy-based augmentation improved the CNN’s ability to recognize important characteristics that were previously hard to pick out. Similar to this, the CNN trained on pictures improved by the standard deviation had remarkable performance, with the best accuracy of 96.1% and AUC of 98.7% among the individual enhancement techniques. By highlighting regions of variability in the picture, standard deviation augmentation enables the model to identify distinguishing characteristics. The model’s high F1-score of 98.9% and accuracy of 95.4% were attained, but its recall of 94.0% was somewhat lower than that of the fuzzy entropy and histogram models, indicating some conservatism in the identification of positive instances. The concatenated CNN performed the best overall by combining characteristics from all three improvement techniques. It achieved a 98.9% accuracy rate, 99.3% precision, 99.8% F1-score, and 99.6% recall. The AUC of 98.7% provided additional evidence of its capacity to successfully differentiate across groups. The concatenated model achieved the maximum performance across all criteria by utilizing the advantages of each individual improvement strategy by incorporating features from the upgraded datasets.

To evaluate the proposed fuzzy enhancement approach, the original dataset was filtered using the CLAHE algorithm and the datasets were evaluated using the BRISQUE algorithm. Additionally, our CNN model was trained on CLAHE-based dataset and compared with our proposed fuzzy-enhanced algorithm-based CNN models as shown in Table 3.

The experimental results, which are shown in Table 1, offer a thorough analysis of several CNN models that were trained on datasets that had been enhanced using a variety of image processing methods, such as fuzzy entropy, histogram equalization, CLAHE (Contrast Limited Adaptive Histogram Equalization), and standard deviation-based enhancement. The BRISQUE (Blind/Referenceless Image Spatial Quality Evaluator) technique was used to measure the quality of the dataset. A CNN model was then used to test the classification accuracy of each upgraded dataset. A summary of the findings is given below, with particular attention on how BRISQUE measures picture quality and how various improvement techniques affect CNN model performance.

With a moderate BRISQUE score of 26.8, the CLAHE algorithm, which aims to improve local contrast, identified some distortions in the images. In spite of this, the CNN that was trained using the CLAHE-enhanced dataset did rather well, with an accuracy of 95.1%, perhaps thanks to better contrast. The greater BRISQUE score, however, indicates that the image quality was subpar. A BRISQUE rating of 22.4 indicates that the dataset produced by the histogram equalization approach has greater visual quality. With 96.9% accuracy, the CNN that was trained on this dataset outperformed the others. Histogram equalization enhanced feature extraction by dispersing pixel intensities and improving global contrast, which led to superior classification performance as compared to the CLAHE-based model. The CNN trained on the fuzzy entropy-based dataset performed the best, with an accuracy of 97.8%. This dataset also had the highest picture quality, with the lowest BRISQUE score of 21.1. Fuzzy entropy improved feature representation and produced better classification results by highlighting minor changes and fine features. Compared to the other approaches, this one produced cleaner pictures, which improved CNN performance. The dataset generated via standard deviation-based augmentation had a BRISQUE value of 22.9, which was comparable to the dataset generated by the histogram-based method. A 96.1% accuracy was attained by the CNN that was trained using this dataset. This approach emphasizes visual variability, although it may be less accurate than the histogram and fuzzy entropy-enhanced models due to noise, as seen by the somewhat larger BRISQUE score.

**Figure 10:**
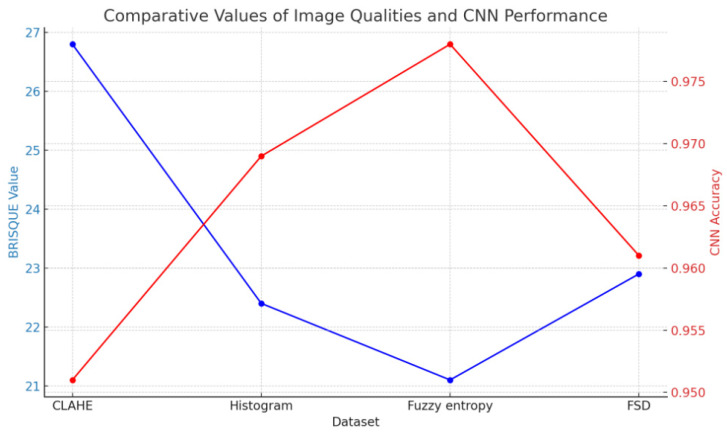
Comparative values of image qualities and CNN performance.

The outcomes and Figure 10 show a strong correlation between CNN model performance (it is indicated with red color) and image quality as determined by the BRISQUE values (it is indicated with blue color). In general, datasets with lower BRISQUE values, a sign of better image quality, had higher classification accuracy. The best overall strategy was revealed to be the fuzzy entropy-based improvement method, which produced the best-quality dataset with the lowest BRISQUE value and the maximum accuracy of 97.8%. This implies that the fuzzy entropy method helps the CNN extract pertinent information for classification tasks while simultaneously improving image quality.

While the accuracy of the CLAHE-based dataset was 95.1%, it correlated with inferior image quality, as indicated by its highest BRISQUE score. Although CLAHE works well for boosting contrast, it might not have as much of an influence on overall image quality as the other techniques. The results show how crucial image enhancing methods are to raising CNN’s level of performance. The suggested fuzzy entropy-based method demonstrated its effectiveness in generating high-quality datasets that result in improved classification outputs, outperforming conventional techniques like histogram equalization and CLAHE.

Table 4 presents the performance of the Concatenated CNN model compared to other neural network models. The assessment measures, which give a thorough picture of each model’s classification skills, include accuracy, precision, F1-score, AUC, and recall. Almost all indicators show that the suggested CCNN model performs better overall, and it stands out with the highest values.

**Figure 11:**
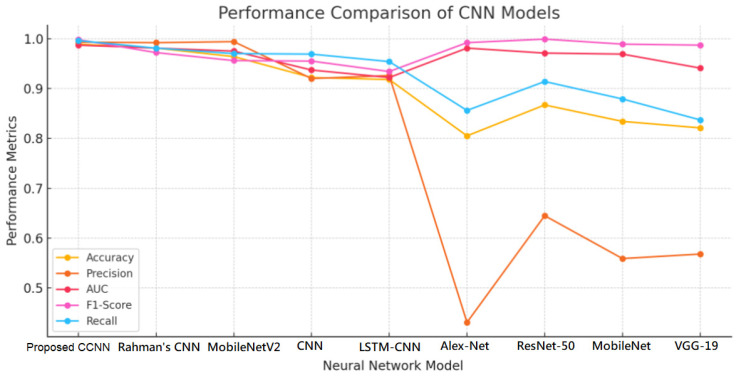
Performance comparison of CNN models.

The Figure 11 demonstrates that, the CCNN performs the best, attaining the greatest recall (99.6%), F1-score (99.8%), accuracy (98.9%), and precision (99.3%). Its remarkable capacity to differentiate across classes is further demonstrated by its AUC (98.7%). The exceptional performance of the CCNN is partly attributed to its design, which combines several feature sets. The almost flawless F1-score shows the optimal ratio of recall to accuracy, reducing false positives and false negatives. Rahman T. et al.’s model has an accuracy of 98.1%, precision of 99.2%, and AUC of 98.1%, which is quite near to the CCNN [40]. The model exhibits strong performance in all measures; however, its recall (98.1%) and F1-score (97.2%) are somewhat lower than those of the CCNN, indicating that the CCNN is more adept at accurately recognizing both positive and negative cases. At 99.4% precision and 96.4% accuracy, MobileNetV2 exhibits impressive performance. Nonetheless, in comparison to the CCNN, its recall (97.0%) and F1-score (95.6%) are lower, suggesting that it could overlook some genuine positives. Despite its high level of precision, this model does not have the recall and precision balance that is required to reduce classification errors, especially in sensitive applications. Moderate performance is achieved by the CNN and LSTM-CNN models, which have accuracy ratings of 92.2% and 91.8%, respectively. Although they are respectable, their F1-scores (95.5% and 93.4%) are not as good as the CCNN’s higher classification performance. While these models work well, they are not as good as more sophisticated designs such as the CCNN.

AlexNet performs far worse, with an accuracy of 80.5% and a very poor precision of 43.1%. Its poor accuracy suggests that it generates a significant percentage of false positives, although having a respectable AUC (98.1%), which renders it untrustworthy for this job. Compared to AlexNet, ResNet-50 performs better, with an F1-score of 99.9% and an accuracy of 86.7%. But when contrasted with the more balanced CCNN, its lower accuracy (64.5%) and recall (91.4%) show inconsistent results when it comes to recognizing true positives. MobileNet and VGG-19 are less competitive because of their lower precision scores (55.9% and 56.8%) and lower accuracy ratings (83.4% and 82.1%, respectively). Even with respectable AUC values (96.9% and 94.1%), these models fall short of the CCNN’s level of accuracy and recall.

In most important performance criteria, the suggested CCNN performs better than the alternatives, according to the examination of Table 2. This challenge finds that the CCNN is the most robust model with the best accuracy, precision, and F1-score. The CCNN outperforms other models in the area by harnessing the advantages of many image enhancing approaches. Because it offers advantages above conventional CNN structures and models from the literature, this places the CCNN as a cutting-edge method for difficult classification problems.

The integration of advanced sensor technology in medical imaging presents a promising pathway to enhance the performance of the CCNN model for pneumonia detection. Modern imaging sensors are becoming increasingly capable of capturing higher-resolution images with greater sensitivity, enabling the detection of fine-grained features in chest X-rays or CT scans. These improvements provide the CCNN model with more detailed data to work with, thereby enhancing its ability to identify pneumonia cases, particularly those that exhibit subtle or early-stage symptoms. In addition to higher resolution, the development of intelligent sensors with real-time feedback mechanisms can dynamically adjust imaging parameters such as contrast, exposure, and noise reduction during the image acquisition process. This ensures that images are of optimal quality before being fed into the CCNN model, minimizing issues related to poor image quality that could affect the model’s diagnostic accuracy. By integrating AI-based image quality assessment tools, these sensors can further automate the process, ensuring consistent and high-quality data collection in various clinical settings, including those with limited resources.

Incorporating advancements in sensor technology with the CCNN model could lead to more accurate and efficient pneumonia detection, improving diagnostic outcomes and making this technology more applicable across diverse healthcare environments. This synergy between cutting-edge sensors and AI models holds great potential for revolutionizing medical imaging and early disease detection.

## 5. Conclusions

The primary innovation of this study lies in the integration of fuzzy logic-based image enhancement with deep learning models, specifically a Concatenated CNN (CCNN) architecture, which offers significant improvements over traditional image processing and deep learning approaches. This hybrid method effectively combines the strengths of fuzzy logic (handling uncertainty and improving image quality) and CNNs (robust feature extraction and classification), resulting in notable gains in performance metrics like accuracy, precision, F1-score, and recall.

1. Fuzzy Logic-Based Image Enhancement: The fuzzy entropy, fuzzy standard deviation, and histogram spread techniques used in this study are novel in their ability to optimize contrast and highlight subtle image features. These methods enable the CCNN to capture fine-grained details that are often missed by traditional enhancement techniques like CLAHE. Unlike previous models that rely on raw or slightly enhanced images, our approach systematically refines image quality using a custom-designed fuzzification refinement algorithm. This leads to significant improvements in the dataset’s visual quality, as measured by the BRISQUE scores, and enhances the CCNN’s ability to detect complex pneumonia cases.

2. Concatenated CNN Architecture: The proposed CCNN model is unique in that it incorporates three distinct input branches corresponding to different image enhancement methods (fuzzy entropy, standard deviation, and histogram equalization). This multi-branch design enables the model to combine complementary features from each enhancement method, leading to higher classification accuracy. While traditional CNN models focus on single datasets, the CCNN’s ability to leverage multiple enhanced datasets is a key differentiator. This structure allows for greater resilience and robustness in classification, particularly in distinguishing between normal and pneumonia cases across diverse image conditions.

3. Comparison with Existing Methods: Most previous studies, such as those using pre-trained CNNs (ResNet, VGG, MobileNet), depend on transfer learning and global image enhancement methods. In contrast, our fuzzy enhancement-based CCNN is trained from scratch, using fuzzy logic to enhance image quality at the pixel level. This approach directly addresses issues related to low-quality or noisy medical images, making the model more effective in clinical environments where image quality can vary. By demonstrating superior performance across multiple datasets and outperforming state-of-the-art models in terms of accuracy, precision, and recall, this study establishes the CCNN as a more versatile and adaptable solution for pneumonia detection. The combination of these enhancement methods in the CCNN framework results in a notable increase in accuracy (98.9%), precision (99.3%), F1-score (99.8%), and recall (99.6%), outperforming alternative models like AlexNet, ResNet-50, and MobileNetV2.

4. BRISQUE-Driven Image Quality Assessment: Highlights that the BRISQUE image quality assessment adds a distinctive dimension to this work. Many existing models focus solely on classification accuracy, but incorporating BRISQUE allows for an objective measure of image quality, directly linking it to CNN performance. This aspect sets our work apart by demonstrating that models trained on datasets with better image quality (lower BRISQUE scores) also achieve higher accuracy, offering a quantitative assessment of the relationship between image quality and CNN performance.

In conclusion, this research introduces a novel combination of fuzzy logic and deep learning that offers improved accuracy, robustness, and diagnostic reliability compared to previous methods. This hybrid approach addresses the limitations of traditional image enhancement techniques, setting a new benchmark for pneumonia detection in medical imaging.

In future work, the role of intelligent sensors can be expanded by integrating them into automated clinical decision support systems, providing real-time feedback during the image acquisition process. By leveraging advances in sensor technology, the CCNN model could be further optimized for edge computing applications, enabling early and efficient pneumonia detection in resource-constrained environments.

## Figures and Tables

**Table 1:** Information about parameters of the proposed CCNN model.

Layer	Input 1	Input 2	Input 3
Convolutional Layer 1:	64 filters, 3 × 3 kernel, ReLU activation		
Max Pooling Layer 1:	2 × 2 pooling, stride = 2		
Convolutional Layer 2:	128 filters, 3 × 3 kernel, ReLU activation		
Max Pooling Layer 2:	2 × 2 pooling		
Convolutional Layer 3:	256 filters, 3 × 3 kernel, ReLU activation		
Max Pooling Layer 3:	2 × 2 pooling, resulting in feature map size 64 × 64 × 256		
Concatenation Layer	Concatenate the feature maps from all three branches along the last axis.		
	Final feature map size: 64 × 64 × 768 (256 from each branch).		
Convolutional Layer 4:	512 filters, 3 × 3 kernel, ReLU activation.		
Max Pooling Layer 4:	2 × 2 pooling, reducing the feature map size to 32 × 32 × 512.		
Convolutional Layer 5:	1024 filters, 3 × 3 kernel, ReLU activation.		
Max Pooling Layer 5:	2 × 2 pooling, reducing the feature map size to 16 × 16 × 1024.		
Flatten Layer:	Flatten the 3D feature map to a 1D vector.		
Dense Layer 1:	1024 neurons, ReLU activation.		
Dropout Layer:	Dropout rate = 0.5		
Dense Layer 2:	512 neurons, ReLU activation.		
Dropout Layer 2:	Dropout rate = 0.5.		
Output Layer	Two neurons (pneumonia or normal), SoftMax activation to assign probabilities.		

**Table 2:** Performance of CNN models.

Neural Network	Accuracy	Precision	AUC	F1-Score	Recall
Original image-based CNN	0.871	0.865	0.847	0.873	0.852
Histogram-based CNN	0.969	0.947	0.935	0.978	0.975
Fuzzy entropy-based CNN	0.978	0.973	0.967	0.979	0.961
Standard deviation-based CNN	0.961	0.954	0.987	0.989	0.940
Concatenated CNN	0.989	0.993	0.987	0.998	0.996

**Table 3:** BRISQUE values of enhanced datasets.

Dataset	BRISQUE Value	Neural Network	Accuracy
CLAHE-based Dataset	26.8	CLAHE-based CNN	0.951
Histogram-based Dataset	22.4	Histogram-based CNN	0.969
Fuzzy entropy-based Dataset	21.1	Fuzzy entropy-based CNN	0.978
FSD-based Dataset	22.9	FSD-based CNN	0.961

**Table 4:** Performance of our Concatenated CNN model with alternatives.

Neural Network Model	Accuracy	Precision	AUC	F1-Score	Recall
Proposed CCNN	0.989	0.993	0.987	0.998	0.996
Rahman T. et al. [43]	0.981	0.992	0.981	0.972	0.981
MobileNetV2 [44]	0.964	0.994	0.975	0.956	0.970
CNN [44]	0.922	0.920	0.937	0.955	0.969
LSTM-CNN [44]	0.918	0.926	0.922	0.934	0.954
AlexNet [45]	0.805	0.431	0.981	0.992	0.856
ResNet-50 [45]	0.867	0.645	0.971	0.999	0.914
MobileNet [45]	0.834	0.559	0.969	0.989	0.879
VGG-19 [45]	0.821	0.568	0.941	0.987	0.837

## Data Availability

Data are contained within the article.
